# Dynamic Changes in the Microbiome and Mucosal Immune Microenvironment of the Lower Respiratory Tract by Influenza Virus Infection

**DOI:** 10.3389/fmicb.2019.02491

**Published:** 2019-11-01

**Authors:** Liming Gu, Huixiong Deng, Zhihui Ren, Ying Zhao, Shun Yu, Yingzhu Guo, Jianping Dai, Xiaoxuan Chen, Kangsheng Li, Rui Li, Gefei Wang

**Affiliations:** ^1^Department of Microbiology and Immunology, Shantou University Medical College, Shantou, China; ^2^Guangdong Provincial Key Laboratory of Infectious Diseases and Molecular Immunopathology, Shantou University Medical College, Shantou, China; ^3^Department of Anesthesiology, The Second Xiangya Hospital of Central South University, Changsha, China

**Keywords:** influenza virus, microbiota, lower respiratory tract, metabolites, transcriptome, mice

## Abstract

Influenza is a major public health concern, and the high mortality rate is largely attributed to secondary bacterial infections. There are several mechanisms through which the virus increases host susceptibility to bacterial colonization, but the micro-environment in lower respiratory tract (LRT) of host, infected with influenza virus, is unclear. To this end, we analyzed the LRT microbiome, transcriptome of lung and metabolome of bronchoalveolar lavage fluid (BALF) in mice inoculated intra-nasally with H1N1 to simulate human influenza, and we observed significant changes in the composition of microbial community and species diversity in the acute (7 days post inoculation or dpi), convalescent (14 dpi) and the recovery (28 dpi) periods. The dominant bacterial class shifted from *Alphaproteobacteria* to *Gammaproteobacteria* and *Actinobacteria* in the infected mice, with a significant increase in the relative abundance of anaerobes and facultative anaerobes like *Streptococcus* and *Staphylococcus*. The dysbiosis in the LRT of infected mice was not normalized even in the recovery phase of the infection. In addition, the infected lung transcriptome showed significant differences in the expression levels of genes associated with bacterial infection and immune responses. Finally, the influenza virus infection also resulted in significant changes in the metabolome of the BALF. These alterations in the microbiome, transcriptome, and metabolome of infected lungs were not only appeared at the acute period, but also observed at the recovery period. Furthermore, the infection of influenza virus induced a long-term effect in LRT micro-environmental homeostasis, which may give a chance for the invasion of potential pathogens.

## Introduction

Influenza is a major public health concern, and the primary cause of death among the critically ill patients, especially the infants, the elderly, pregnant women and those with chronic infection, is secondary bacterial infection. Almost 95% of the 50 million deaths that occurred during the Spanish flu outbreak were the result of secondary bacterial infections (Morens et al., 2008). The influenza virus weakens the host immune responses and increases the risk of secondary infections through several mechanisms, including disruption of the mucosal barrier function in the respiratory tract that increases bacterial adhesion, impairing macrophage and neutrophil function, and inducing interferon type I that inhibits the production of antimicrobial peptides. Although studies have explored several molecular and cellular mechanisms underlying secondary bacterial infection post IAV infection, the role of lung microflora has been largely ignored. Next-generation sequencing has shown bacterial colonization in the pulmonary cavity (Charlson et al., 2011), contradicting the traditional view that the lungs are sterile (Laurenzi et al., 1961; Jordan et al., 1976; Baughman et al., 1987; Thorpe et al., 1987). Recent studies have reported that the alteration of lower respiratory tract (LRT) microbiome is associated with diseases. For example, patients with cystic fibrosis (CF) (Muhlebach et al., 2018), idiopathic pulmonary fibrosis (Molyneaux et al., 2014) and bronchiectasis (Tunney et al., 2013; Lee et al., 2018) had significant increase of bacteria in LRT. In addition to the changes in microbial load, specific bacteria can be detected in the respiratory tract of different diseases, including *Pseudomonas aeruginosa*, *Staphylococcus aureus* or *Burkholderia* spp. (in the case of CF) (Huang and LiPuma, 2016; Ubags and Marsland, 2017); *Haemophilus*, *Veillonella*, *Streptococcus* or *Neisseria* (in idiopathic pulmonary fibrosis) (Fastres et al., 2017; Hewitt and Molyneaux, 2017); or *P. aeruginosa*, *Veillonella*, *Prevotella* or *Haemophilus* (in bronchiectasis) (Tunney et al., 2013; Lee et al., 2018). Therefore, the micro-environmental homeostasis of LRT is important for the defense against the invasion of potential pathogens. However, the micro-environment of LRT is unclear on the condition of influenza virus infection, especially on long-term observation.

The objective of this study was to determine host anti-virus activity, microbial flora and metabolic mass in the LRT of mice during influenza virus infection. To this end, we compared the lung microbiome, transcriptome, and metabolome of mice inoculated with influenza virus or saline, and observed dynamic changes in the LRT microbiome during the different periods of infection. In addition, transcriptome sequencing and non-target metabolomic analysis of the alveolar bronchoalveolar lavage fluid (BALF) were also conducted to analyze the local immune microenvironment.

## Results

### Systemic Changes After Influenza Virus Infection

**FIGURE 1:**
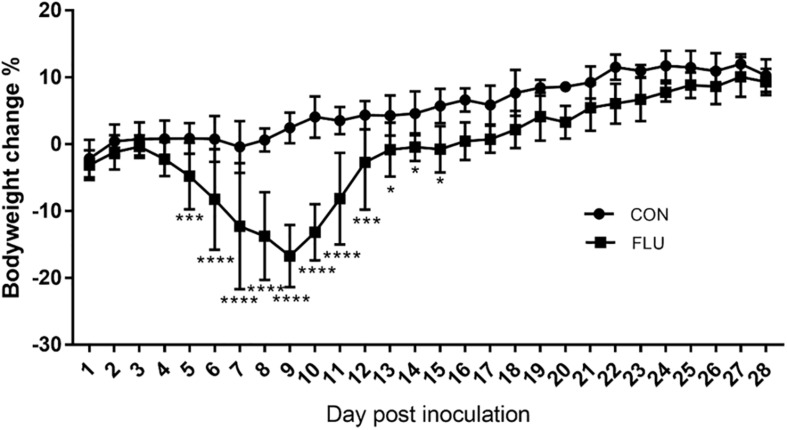
Influenza-infected mice (FLU, *n* = 6) lost a significant amount of weight from 3 to 12 dpi compared to mice inoculated with saline (CON, *n* = 6). Data are presented as means ± SD (^∗^*p* < 0.05, ^∗∗∗^*p* < 0.001, ^****^p < 0.0001 compared with CON; Same as the followed text).

Weight loss is an established index of the immune response to influenza (Matsuoka et al., 2009), and as shown in Figure 1, the body weight of the mice infected with the non-neurotropic H1N1/PR8 virus decreased significantly in a time-dependent manner compared to the uninfected controls from day 5 to 15 post inoculation. It began to decrease from 4 days post inoculation (dpi) and was significantly lower at 9 dpi (−16.72%, *p* < 0.0001), and increased steadily from 10 dpi to 16 dpi (0.47%, *p* = 0.0803). While the uninfected mice showed no obvious change in activity or behavior, and had bright and smooth fur, the infected mice started showing signs of listlessness, aggregation, tremors, lackluster fur, and kyphosis from 3 dpi.

### LRT Microbiota Diversity of Mice Infected With H1N1

**FIGURE 2:**
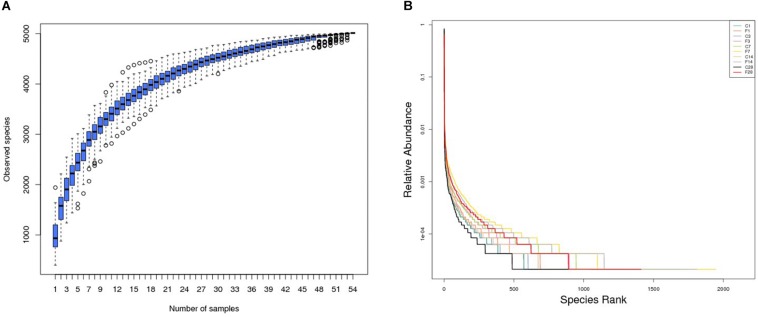
Altered LRT microbiome in mice (*n* = 54) inoculated with saline or H1N1 influenza virus in terms of **(A)** species richness and **(B)** relative abundance (C, the group treated with saline; F, the group treated with influenza virus; the number followed C/F represented the day post inoculation).

The rationality of the sequencing data was determined by the rarefaction curve, and as shown in Supplementary Figure S1, the gradual flattening indicated that the sequencing strategy adopted in this study was reasonable, and inclusion of more sequencing data would only result in a few more operational taxonomic units (OTUs). Furthermore, the species accumulation box plot (Figure 2A) showed that increase in sample size did not increase species diversity and richness, indicating that our sample size was adequate and the annotated species were representative of the microbiota.

**FIGURE 3:**
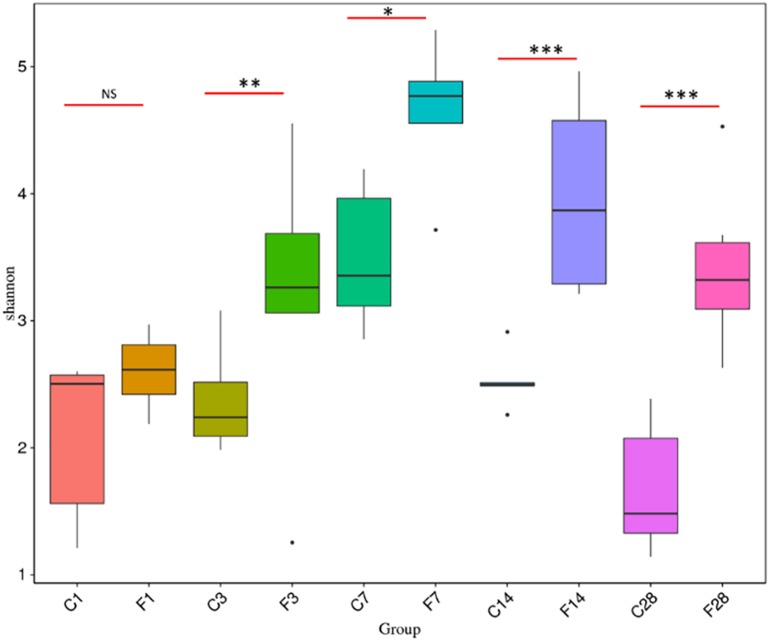
Comparison of Shannon Index based on the genera profile in different groups (C, the group treated with saline; F, the group treated with influenza virus; the number followed C/F represented the day post inoculation). There were 5–6 mice in each group, and each sample was analyzed independent (^∗^*p* < 0.05, ^∗∗^*p* < 0.01, and ^∗∗∗^*p* < 0.001, NS no significant compared with CON).

The rank abundance curve (Lundberg et al., 2013) shown in Figure 2B indicated high species richness (increased width in the horizontal axis) in the LRT but uneven distribution (steep gradient in the *Y*-axis). The species diversity was evaluated using the Shannon index and compared across different groups with the Wilcox rank test. As shown in Figure 3 and Supplementary Table S1, the species diversity of the uninfected controls at 7 dpi was significantly higher than at 1 dpi, while no significant difference was seen among the other time points. In the infected animals, the LRT species diversity was significantly higher compared to that of the controls at 3, 7, 14 and 28 dpi. Within the group, the species diversity was the highest at 7 dpi, decreased slightly at 14 dpi, and was significantly lower at 28 dpi (*p* = 0.0128 compared to 7 dpi). Therefore, the diversity of the LRT microbial flora increased rapidly in the acute period of the infection, and decreased during the convalescent and recovery periods. Nevertheless, the species diversity didn’t restore to the normal levels even after 28 dpi.

### Structure of Bacterial Communities

**FIGURE 4:**
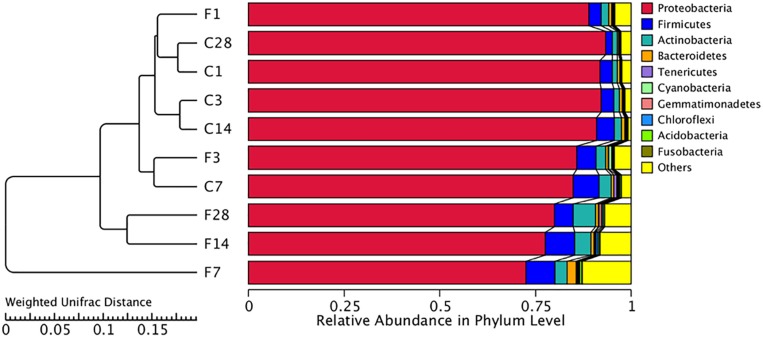
Unweighted pair-group method with arithmetic mean (UPGMA) cluster tree based on weighted uniFrac distance (left) and the relative abundance profiles of species at the phylum level (right). The majority of the OTUs were dominated by ten phyla: *Proteobacteria*, *Firmicutes*, *Actinobacteria*, *Tenericutes*, *Cyanobacteria*, *Gemmatimonadetes*, *Chloroflexi*, *Acidobacteria*, and *Fusobacteria*. Based on the weighted uniFrac distance, the microbiome of LTR treated with influenza virus at the 7, 14, and 28 dpi are similar, and far different from saline-treated group. C, the group treated with saline; F, the group treated with influenza virus; the number followed C/F represented the day post inoculation.

The abundance of different microbial phyla in murine lungs shows considerable variations during development, indicating a dynamic microbial ecosystem (Singh et al., 2017). However, it stabilizes during adulthood, and is dominated by the Proteobacteria, Firmicutes, Actinobacteria and Bacteroidetes phyla. The microbial community structure of different samples was compared using the weighted uniFrac distance matrix and unweighted pair-group method with arithmetic mean (UPGMA) cluster analysis, which indicated similar abundance at the phylum level (Figure 4). While F1, F3, and CON (all time points) groups showed high similarity, the community structure of F14 was more similar with that of F28. The F7 group was the farthest from the other groups, indicating a unique microbial community structure. Similar results were obtained from the principal coordinate analysis (PCoA) analysis (Supplementary Figure S2).

The non-parametric ANOSIM test was then used to determine whether the inter-group differences were significantly greater than the intragroup differences. As shown in Table 1, the intragroup difference was greater at 1 dpi and 3 dpi (*R* < 0), whereas the intergroup difference was greater at 7, 14, and 28 dpi (*R* > 0).

**TABLE 1:** Comparison of the rank of the distance between samples by ANOSIM test.

**Group**	***R*-value**	***p*-value**
C1-F1	0.012	0.449
C3-F3	0.176	0.062
C7-F7	0.208	0.044
C14-F14	0.4853	0.009
C28-F28	0.463	0.007

**FIGURE 5:**
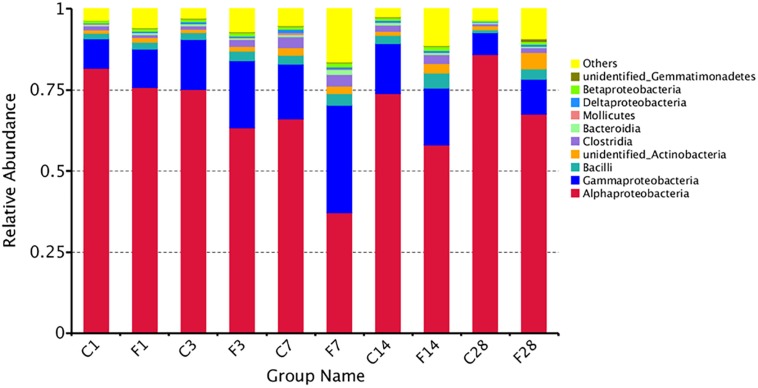
The relative abundance of microbial communities at the level of class (C, the group treated with saline; F, the group treated with influenza virus; the number followed C/F represented the day post inoculation). The majority of the OTUs were dominated by ten classes: *Alphaproteobacteria*, *Gammaproteobacteria*, *Bacilli*, *Unidentified Actinobacteria*, *Clostridia, Bacteroidia*, *Mollicutes*, *Deltaproteobacteria*, *Betaproteobacteria*, and *Unidentified Gemmatimonadetes*. Identities were established using sequence homology with 16S rRNA gene sequences. Different proportions of classes can be seen at different day post inoculation of the LRT. More than 85% of the reads belonged to these ten classes.

**FIGURE 6:**
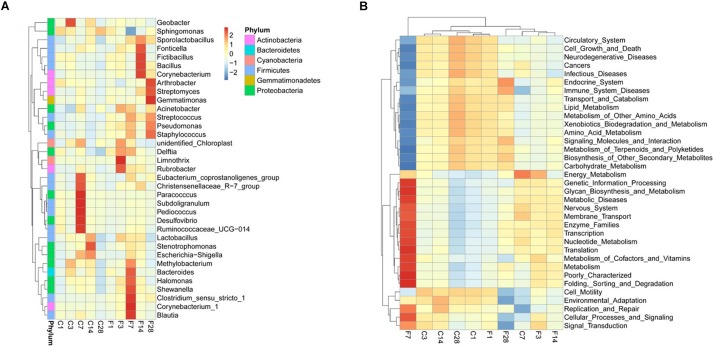
Composition and genomic function of LRT microbiota after influenza virus infection showing cluster heat maps of **(A)** species abundance at the genus level and **(B)** genomic function (C, the group treated with saline; F, the group treated with influenza virus; the number followed C/F represented the day post inoculation. *n* = 5 or 6 per group).

The top 10 relatively abundant classes in the uninfected LRT were *Alphaproteobacteria, Gammaproteobacteria, Bacilli, unidentified Actinobacteria, Clostridia, Bacteroidia, Mollicutes, Deltaproteobacteria, Betaproteobacteria* and *unidentified Gemmatimonadetes* (Figure 5). The dominant class was *Alphaproteobacteria*, with >80% abundance relative to the infected mice. Following infection of influenza virus, the relative abundance of *Alphaproteobacteria* decreased and that of *Gammaproteobacteria* increased during the acute period, indicating a shift in the dominant class. The top 35 genera were shown in the heat map in Figure 6A, and included *Actinobacteria, Bacteroidetes, Cyanobacteria, Firmicutes, Gemmatimonadetes* and *Proteobacteria* among others. *Geobacter* and *Methylobacterium* were more abundant in the control group at 3 dpi, *Eubacterium coprostanoligenes group, Christensenellaceae R-7 group, Paracocccus, Subdoligranulum, Pediococcus, Desulfovibrio, Ruminococcaceae UCG-014* and *Escherichia-Shigella* at 7 dpi, and *Lactobacillus, Stenotrophomonas* and *Escherichia-Shigella* at 14 dpi. In the infected group, *Acinetobacter*, *unidentified Chloroplast*, *Delftia, Limnothrix* and *Rubrobacter* were more abundant at 3 dpi, *Acinetobacter, Streptococcus, Pseudomonas, Staphylococcus, Delftia, Methylobacterium, Bacteroides, Halomonas, Shewanella, Clostridium sensu stricto1, Corynebacterium-1* and *Blautia* at 7 dpi, *Sporolactobacillus*, *Fonticella, Fictibacillus, Bacillus* and *Corynebacterium* at 14 dpi, and *Arthrobacter*, *Streptomyces*, *Gemmatimonas*, *Streptococcus, Pseudomonas* and *Staphylococcus* at 28 dpi. During the acute period of infection, the relative abundance of *Sphingomonas* decreased in the LRT, while that of the facultative anaerobes and anaerobes increased. The genomic functions of the microbiota were predicted using PICRUSt software based on the 16S V4 rRNA sequencing data, and were found to be altered upon influenza virus infection, especially at 7 dpi (Figure 6B). The functions of cell motility and environmental adaptation decreased at 28 dpi, indicating decreased ability of the LRT microbiota to control any pathogenic invasion.

### Changes in Lung Gene Expression Following Influenza Infection

**FIGURE 7:**
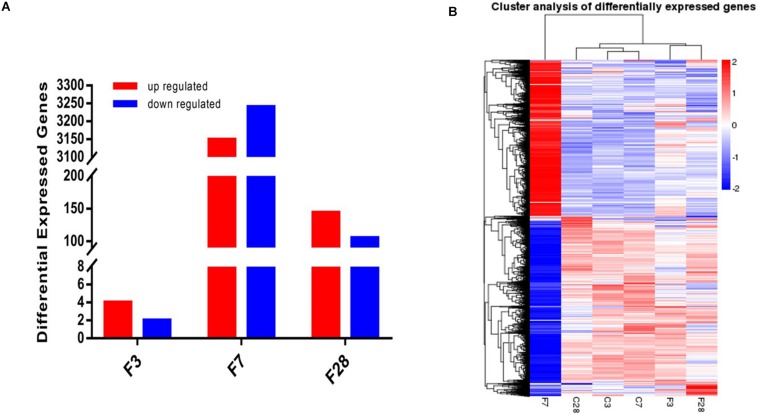
Whole transcriptome analysis of lungs exposed to influenza virus/saline showing **(A)** 6, 6389 and 249 DEGs in the H1N1-infected mice on 3, 7, and 28 dpi, and **(B)** gene clustering based on expression levels (FPKM). Blue color indicates the low-expressed-level genes and red color the high-expressed-level genes in the whole data set (C, the group treated with saline; F, the group treated with influenza virus; the number followed C/F represented the day post inoculation. *n* = 3 per group).

Prior to the transcriptome sequencing, we tested the correlation of gene expression levels between samples. The Pearson correlation coefficient *R*^2^ of the biological repetition interval (*n* = 3) in each group was greater than 0.95 (Supplementary Figure S3), which is higher than the recommended 0.92 by ENCODE, indicating highly similar expression patterns across samples. Six differentially expressed genes (DEGs), including 4 up- and 2 down-regulated genes, were identified in the precursory stage, 6389 (3149 up- and 3240 down-regulated) in the acute stage and 249 (144 up- and 105 down-regulated) in the recovery stage (Figure 7A). Cluster analysis on the screened DEGs showed similar expression patterns of C3, C7, and C28 (control) groups, and of the F3 and F28 groups. The expression pattern of the F7 group was significantly different from the others (Figure 7B).

**FIGURE 8:**
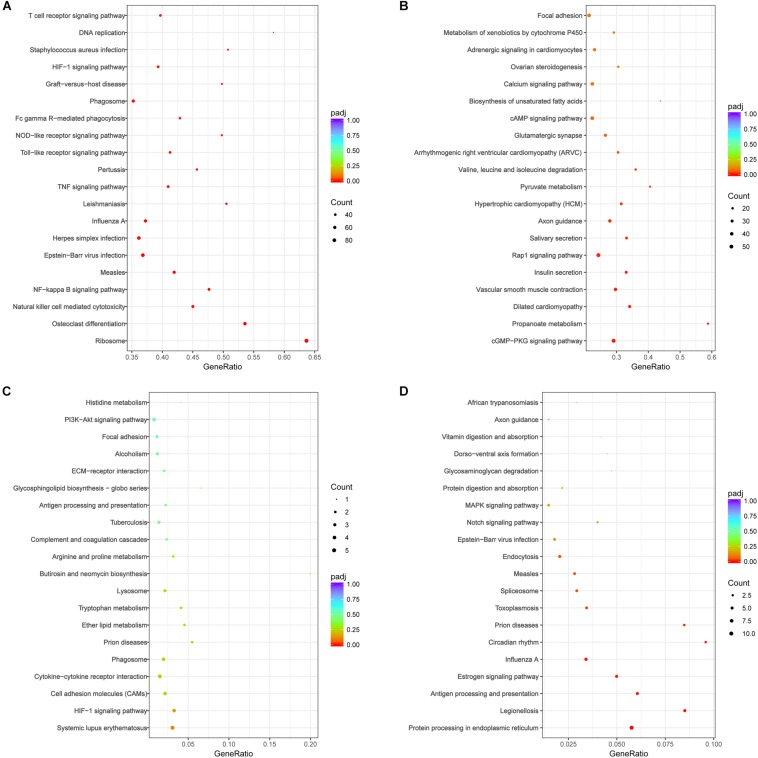
KEGG pathway analysis of DEGs following H1N1 infection revealed significant pathways (*p*adj < 0.05) involved in local immune responses and cell adhesion molecules in the lung of mice on **(A,B)** 7 and **(C,D)** 28 dpi. **(A)** The enrichment pathways of up-regulated genes at 7 dpi; **(B)** the enrichment pathways of the down-regulated genes at 7 dpi; **(C)** the enrichment pathways of up-regulated genes at 28 dpi; **(D)** the enrichment pathways of down-regulated genes at 28 dpi. The size of the circle indicated the number of the genes. The color of the circle indicated *Q* value. Gene Ratio refers the ratio of Sample number and Background number enriched in the pathway. The value range of adjusted *P*-value (*p*adj) is [0,1], and the closer to zero, the enrichment is more significant.

The DEGs were performed analysis of KEGG signal pathway enrichment by hypergeometric test/Fisher’s exact test and adjusted by Benjamini and Hochberg (*p*adj value<0.05). KEGG pathway enrichment analyses revealed that the DEGs in the acute phase (7 dpi) were significantly enriched in immune response, response to stress, positive regulation of biological process, and intracellular and protein binding function. The inflammatory pathways of Toll-like receptor signaling, TNF signaling and T cell receptor signaling, as well as pathways related to infectious disease and immune clearance, such as *S. aureus* infection, measles, pertussis, leishmaniasis, influenza A, phagosome, natural killer cell mediated cytotoxicity, and Fc gamma R-mediated phagocytosis, were up-regulated in the acute phase (Figure 8A). In contrast, the DEGs associated with functions like morphogenesis, cellular or subcellular motion and ciliary movement, pathways of vascular smooth muscle contraction, salivary secretion and biosynthesis of unsaturated fatty acids were decreased (Figure 8B). Taken together, the genes associated with anti-pathogen response were upregulated, and those involved in homeostasis were downregulated in the lungs during the acute phase of influenza infection.

Fewer DEGs were observed during the recovery stage, and were enriched in the functions of immune system process, response to stress, regulation of response to stimulus, positive regulation of biological process and extracellular region expression. However, no significant enrichment of up-regulated genes was seen in the KEGG signaling pathways (adjusted *P*-value > 0.1), as shown Figure 8C. The downregulated DEGs were enriched in functions of macromolecule metabolic process, regulation of nucleobase-containing compound metabolic process, regulation of nitrogen compound metabolic process, DNA-template and unfolded protein binding, and significantly enriched in the pathways of protein processing in endoplasmic reticulum, antigen processing and presentation, toxoplasmosis, measles, prion diseases, legionellosis, influenza A, endocytosis, circadian rhythm, estrogen signaling, and spliceosome (Figure 8D). The decrease in the DEGs associated with antigen processing and presentation, endocytosis and the pathways of defense disease may suppress host immunity during the recovery period of infection.

### Metabolic Changes in BALF After Influenza Infection

The BALF extracts were analyzed using LC/QTOFMS, and the PLS-DA models showed a significant difference between saline and infected BALF samples, with 2581 final features. The distinct metabolites between uninfected and infected BALF were screened using VIP >1 and *p* < 0.05 as the criteria, resulting in 22 altered metabolites in the influenza samples at the prodromal phase, 39 at the acute phase and 9 at recovery phase. The prodromal phase metabolites included lysophosphatidic acid (LPA), cholesterol, D-Mannitol, L-Valine, uric acid, L-Methionine, shikimate-3-phosphate, PG (12:0/13:0), S-Adenosylhomocysteine, phosphoribosylamine, α-D-Glucose, gluconolactone, histamine, dihydroorotic acid, adenosine thiamine triphosphate, L-Homocysteic acid, UDP-glucose, 3-chlorobenzoic acid, and shikimic acid (Table 2). LPA is previously associated with lung injury (Georas et al., 2007; Tager et al., 2007; Zhao et al., 2009, 2011). At the acute phase, the altered metabolites included psychosine, progesterone, N1-Acetylspermine, cortisol, gluconolactone, citric acid, chloroacetic acid, β-hydroxypyruvic acid, gulonic acid, levoglucosan, gentisic acid, palmitoyl-L-carnitine, LPA (0:0/18:0), shikimic acid, S-adenosylmethionine, DL-2-aminoadipic acid, sphingosine, oleic acid, D-fructose 1,6-bisphosphate, α-ketoisovaleric acid, citrulline, L-arabitol, acetoacetyl-CoA, d-Dethiobiotin, phosphocholine, hydroxyurea, phosphocreatine, hexylamine, thiamine, sebacic acid, DL-α-Lipoic Acid, flavin mononucleotide (FMN), acitretin, N-acetyl-L-histidine, 2′-deoxyuridine 5′-diphosphate (dUDP), L-carnitine, L-threonine, argininosuccinic acid, and heme (Table 3). LPA, oleic acid, L-carnitine, psychosine, and progesterone are associated with lung injury and protection. At the recovery phase, carnosine, oleic acid, guanidylic acid, D-urobilin, dodecanamide, L-histidine, glutathione oxidized, phosphoribosylamine, and sorbic acid were altered in the infected samples (Table 4). Oleic acid is associated with lung inflammation and injury, carnosine suppresses lipopolysaccharide (LPS)-induced lung injury by reducing ROS activity (Tanaka et al., 2017), and ameliorates the influenza-induced pathological lesions in lungs (Xu et al., 2015).

**TABLE 2:** Differential BALF metabolites on 3 dpi.

**Name**	**VIP**	**mz**	**rt**	***P***	**Fold change (F/C)**
LPA (0:0/18:0)	2.216	439.298	11.064	0.001	1.299
Cholesterol	1.933	385.376	11.878	0.001	1.254
D-Mannitol	1.746	181.056	17.920	0.006	0.638
L-valine	1.396	152.068	17.790	0.042	0.465
Uric acid	1.622	202.996	16.047	0.013	0.394
L-methionine	1.965	184.012	16.606	0.001	0.235
Shikimate-3-phosphate	1.849	288.994	0.797	0.002	−0.121
PG (12:0/13:0)	1.639	625.407	8.716	0.037	−0.134
S-adenosylhomocysteine	1.380	419.067	18.013	0.045	−0.147
Phosphoribosylamine	1.497	228.035	16.790	0.026	−0.192
α-d-Glucose	1.751	179.052	18.069	0.006	−0.206
Gluconolactone	1.886	177.039	18.087	0.002	−0.207
Histamine	1.785	112.087	18.089	0.020	−0.217
Dihydroorotic acid	1.981	157.022	1.198	0.001	−0.229
L-fucose 1-phosphate	2.015	278.999	18.457	0.000	−0.280
Oxoglutaric acid	1.912	180.994	18.399	0.001	−0.324
Docosanedioic acid	1.851	371.313	5.212	0.014	−0.362
Adenosine thiamine triphosphate	1.542	753.033	0.759	0.021	−0.380
L-homocysteic acid	2.055	182.014	18.387	0.000	−0.384
UDP-glucose	1.570	565.047	18.051	0.018	−0.395
3-chlorobenzoic acid	2.247	154.990	18.402	0.000	−0.478
Shikimic acid	1.602	173.054	0.852	0.015	−0.530

**TABLE 3:** Differential BALF metabolites on 7 dpi.

**Name**	**VIP**	**mz**	**rt**	***p***	**Fold change (F/C)**
Psychosine	1.969	462.392	11.073	0.001	0.580
progesterone	2.005	313.230	16.587	0.001	0.322
N1-Acetylspermine	1.483	245.260	8.565	0.034	0.279
Cortisol	1.848	363.203	13.584	0.004	0.206
Gluconolactone	1.553	177.039	18.087	0.028	0.173
Citric acid	1.479	191.015	18.179	0.039	0.095
Chloroacetic acid	1.527	92.975	0.889	0.031	0.067
β-Hydroxypyruvic acid	1.614	103.008	0.797	0.021	0.066
Gulonic acid	1.406	219.039	18.421	0.047	−0.068
Levoglucosan	1.483	185.043	18.071	0.034	−0.093
Gentisic acid	1.396	155.037	1.390	0.049	−0.121
Palmitoyl-L-carnitine	1.666	400.330	9.822	0.013	−0.168
LPA (0:0/18:0)	1.707	439.298	11.064	0.010	−0.222
Shikimic acid	1.438	173.054	0.852	0.046	−0.270
S-adenosylmethionine	1.490	399.173	4.466	0.033	−0.285
DL-2-aminoadipic acid	1.477	160.062	0.685	0.039	−0.291
Sphingosine	1.581	300.279	4.725	0.021	−0.312
Oleic Acid	1.414	305.236	4.719	0.046	−0.314
D-fructose 1,6-bisphosphate	1.463	341.010	0.741	0.037	−0.328
α-ketoisovaleric acid	1.735	115.050	0.778	0.010	−0.341
Citrulline	1.704	174.082	1.334	0.013	−0.378
L-arabitol	1.943	153.082	0.667	0.002	−0.386
Acetoacetyl-CoA	1.705	850.158	0.759	0.013	−0.436
d-Dethiobiotin	1.575	215.160	5.590	0.022	−0.468
Phosphocholine	1.638	182.060	0.815	0.018	−0.630
hydroxyurea	1.713	99.019	0.982	0.010	−0.653
Phosphocreatine	1.818	234.024	1.296	0.005	−0.680
Hexylamine	2.185	124.110	1.315	0.000	−0.725
Thiamine	1.554	263.095	1.297	0.028	−0.726
Sebacic acid	1.624	201.119	1.315	0.020	−0.838
DL-α-lipoic acid	1.710	205.033	0.982	0.012	−0.890
Flavine mononucleotide (FMN)	2.083	455.073	5.595	0.001	−0.901
Acitretin	1.921	349.171	0.797	0.002	−1.047
N-acetyl-L-histidine	1.934	198.070	1.556	0.002	−1.182
2′-deoxyuridine 5′-diphosphate (dUDP)	1.922	411.001	5.578	0.002	−1.448
L-carnitine	2.088	162.111	1.889	0.000	−1.485
L-threonine	1.832	120.065	1.574	0.004	−1.656
Argininosuccinic acid	1.944	291.149	1.704	0.002	−1.890
Heme	1.508	651.144	5.587	0.034	−2.194

**TABLE 4:** Differential BALF metabolites on 28 dpi.

**Name**	**VIP**	**mz**	**rt**	***p***	**Fold change (F/C)**
Carnosine	2.140	225.104	0.852	0.013	0.583
Oleic acid	2.023	305.236	4.719	0.029	0.322
Guanidylic acid	1.954	385.995	18.070	0.037	0.096
D-Urobilin	1.928	589.340	8.525	0.041	0.080
dodecanamide	1.874	200.223	7.883	0.048	−0.049
L-histidine	2.236	156.090	4.466	0.013	−0.092
Glutathione, oxidized	2.314	647.100	0.778	0.006	−0.156
Phosphoribosylamine	2.181	228.035	16.790	0.011	−0.174
Sorbic acid	2.424	111.053	16.364	0.003	−0.187

To analyze the impact of these altered metabolites on LRT microbiota, we calculated the mathematical correlation using Spearman test, and found a significant relationship (Supplementary Figures S4–S6). During the recovery period (28 dpi), increased oleic acid, decreased L-histidine, and sorbic acid were associated with lower cell motility, and decreased levels of glutathione oxidized, L-histidine and sorbic acid with poor environmental adaptation (Supplementary Figure S6).

## Discussion

The inability of conventional techniques to detect microorganisms in the sputum and lung extracts led to the erroneous belief that the lung cavities are sterile (Laurenzi et al., 1961; Jordan et al., 1976; Baughman et al., 1987; Thorpe et al., 1987; Dickson et al., 2016). Therefore, the lung microbiota is not included in the Human Microbiome Project (Peterson et al., 2009). Next generation sequencing, however, revealed a rich microbial community in the LRT of healthy individuals (Charlson et al., 2011), dominated by *Prevotella, Veillonella, Streptococcus, Fusobacterium*, and *Haemophilus.* In murine models, the LRT microbial community changes significantly from birth till 6 weeks postnatal, and stabilizes by the 8th week (Singh et al., 2017). Consistent with this, the LRT of the 9-weeks-old mice were rich in biological diversity, but had uneven species distribution. The dominant genera were *Sphingomonas*, *Halomonas*, *Shewanella*, and *Acinetobacter*, which belong to the class *Proteobacteria*, and of which *Sphingomonas* was the most abundant (80%). Interestingly, even saline inoculation into the nasal cavity altered the LRT microflora structure, with significant differences seen in the abundance of *Paracoccus marcusii* and *Rhodobacterales* at 7 dpi, and in *Sphingomonas meloni* at 28 dpi. The development of lung microbiome in mice (C57BL/6) is from an early age of 1–6 weeks, and then remained stable at later ages (Singh et al., 2017). Studies show that any liquid in the nostrils can enter the lungs through nasal leak, resulting in migration of the nasal microbes to the pulmonary tract (Porat et al., 1991). Therefore, the transient alteration in the LRT microbial community structure could be the result of the transfer of upper respiratory tract (URT) flora through the nasal leak, which then gradually removed by the LRT immune barriers as well as the inherent microflora. Despite these indirect evidences, there is still a lack of direct evidence to prove that these changes are caused by nasal inoculation. The best solution is to set up another group of naive control without treatment, which is the deficiency of this experiment. Attention should be paid to the influence caused by nasal inoculation in future LRT microbiome research. Whereas, the imbalance of LRT microbiome was not recovery, even at 28 dpi treated with influenza virus infection. There was a transiently alteration of LRT microbiome in the group of saline treatment at 7 dpi, but recovered at 14 dpi. Although there was a shift of microflora in saline-treated group, the alteration was more significant in flu-treated mice compared with saline-treated mice at 7 dpi. The composition of LRT microbiota is mainly affected by migration, elimination and reproduction rate of microorganisms (Dickson et al., 2014; Huffnagle et al., 2017). Therefore, the imbalance of LRT microbiome may be induced by the followed factors. First, the consumption of the host’s immune system is caused by influenza virus infection. For example, the depletion of alveolar macrophages (Ghoneim et al., 2013) weakens the host’s ability to regulate LRT microbiome. Second, as shown in our metabolome data, the microenvironment of LRT changes at 28 dpi, which is also an important factor affecting LRT microbiome.

### Microbial Ecosystem of LRT Is Altered After Influenza Virus Infection

Influenza virus significantly altered the microbial community structure in the LRT, manifested largely by the replacement of *Sphingomonas* by *Halomonas* and *Shewanella* (class *Gammaproteobacteria*) as the predominant genera. The relative abundance of *Sphingomonas* decreased to 35.33% at 7 dpi, compared to 58.69% in the control group. In addition, the proportion of previously low-abundance species also increased from 6.82 to 17.69%. During the acute period of infection, the genera *Halomonas*, *Shewanella*, and *Clostridiales* were the most abundant, while *Bacillales* and *unidentified Actinobacteria* were the most abundant genera in the recovery period. The relative abundance of the *Gammaproteobacteria*, *Firmicutes*, and *Bacteroides* classes increased significantly in the LRT during the acute and recovery period of infection. *Gammaproteobacteria* includes several pulmonary gram-negative pathogens like *Acinetobacter*, *Pseudomonas*, and *Stenotrophomonas*, *Firmicutes* includes facultative anaerobes like *Staphylococcus*, *Streptococcus*, *Lactobacillus*, and so on, and the anaerobe *Prevotella* in *Bacteroidetes* can cause pneumonia. Therefore, the differential species profile can be a potential biomarker for the different stages of influenza virus infection. On the other way, many studies have found that the development of respiratory tract infection and disease is related to the immune regulation of the colonized microorganisms. For example, infants with early LRT infection, changing the structure of original respiratory tract microbial colonization will increase the chances of allergic reactions and repeated wheezing in the future (Kusel et al., 2007; Jackson et al., 2008). In addition to infection, early colonization of the respiratory tract of children with *Streptococcus pneumoniae*, *Moraxella catarrhalis* or *Haemophilus influenzae* can promote the progression of chronic wheezing even if they do not show any symptoms (Hilty et al., 2010; Bisgaard et al., 2011; Teo et al., 2015). In addition, in the treatment of H7N9 patients, microbial regulators can promote the increase of intestinal *Bifidobacterium*, *Lactobacillus*, and *butyric acid-producing bacteria*, which can reduce secondary bacterial infection and improve the survival rate of patients (Lu et al., 2014; Hu et al., 2016). Consistent with our findings, previous studies found significant differences in the LRT microflora of infected individuals through PCoA analysis, possibly due to virus-mediated destruction of the URT barrier which leads to bacterial drift into the lungs (Sajjan et al., 2008; Comstock et al., 2011), resulting in the increased microbial diversity in the lungs and considerable intra-group differences.

In the gastrointestinal tract, an inflammatory environment provides terminal electron receptors to some *Gammaproteobacteria* anaerobes that ferment the substrates in the mucosal layer and alter the metabolic environment (Winter et al., 2013; Winter and Baumler, 2014a, b), and also synthesize pro-inflammatory chemicals. The microbe-associated molecular patterns (MAMPs) on the bacterial surfaces bind to the receptors on immune cells to promote inflammation, exemplified by LPS that can interact with the TLR4 on immune cells (Larsen et al., 2015). This increase of anaerobes and facultative anaerobes in the LRT during influenza could be the result of increased mucus production and disruption of mucosal epithelia, which promotes exudation of fluids and creates an anaerobic environment in the lungs. Furthermore, destruction of the mucosal epithelial barrier and resulting tissue injury and hemorrhage release massive amount of nutrients for (anaerobic) bacterial growth and colonization. The rapidly proliferating anaerobes also lead to significant changes in the microbial community structure during the acute phase of infection, which may not be restored even during the recovery period. While the microbiota of the URT can effectively resist pathogenic invasion, that of the LRT is less resistant due to uneven species distribution and lower complexity. In addition to the changes described above, the host immune response also destroys the original microbial ecosystem and promotes the adherence and proliferation of the immigrant species. Contradictory to our findings, another study found no significant alterations in the LRT microbiota throughout the course of influenza infection (Yildiz et al., 2018), which may be attributed to different virus strains and severity of infection.

At the acute phase (7 dpi), up-regulated genes were enriched in ribosome and DNA replication to activate host immune response (pathways of Toll-like receiver signal, Fc gamma R-mediated phagocytosis, Phagosome, and T cell receiver signaling) and defense against the pathogens via the pathways of measles, Epstein–Barr virus infection, herpes simplex infection, influenza A, leishmaniasis, pertussis, and *S. aureus* infection. At the same time, down-regulated genes were enriched in basic metabolism and communication pathways that maintain physiological homeostasis. For example, focal adhesions (FAs) link the extracellular matrix to the actin cytoskeleton to mediate cell adhesion, migration, and signaling (Case et al., 2015). Cytochrome P450 (P450) enzymes regulate a variety of endogenous signaling molecules and play central roles in the metabolism of xenobiotics and drugs (Wright et al., 2009). At the recovery period (28 dpi), the DEGs were not same as acute phase which the up-regulated genes and enriched in pathways against pathogens invasion, but the down-regulated genes were enriched in disease resistance pathways as influenza A, prion diseases, measles, legionellosis. In addition, the pathways of antigen processing and presentation and endocytosis were also enriched by the downregulated DEGs, which indicated that the host’s ability to resist pathogens invasion is weaken at this time. At the same time, the relative abundance of LRT microbiome, such as *S. aureus*, was a higher level (compared with 1 dpi). In brief, the hosts may not be strong enough to defense against potential pathogens at the recovery phase (28 dpi).

In contrast to the LRT transcriptome and microbiome, the BALF metabolome of the infected mice was significantly altered during the prodromal phase. LPA, a small lipid that mediates various cellular functions via the activation of LPA receptors, increased during the prodromal phase and decreased in the acute phase. It is also known to increase in inflammatory conditions like asthma (Georas et al., 2007; Zhao et al., 2009), fibrosis (Tager et al., 2007), and acute lung injury (Zhao et al., 2011). Oleic acid, a pro-inflammatory unsaturated fatty acid and the biological marker of ARDS severity (Goncalves-de-Albuquerque et al., 2015), decreased in the acute phase and increased in the recovery phase. Heme also decreased in the acute phase, while heme oxygenase (Hmox1 and Hmox2) which is induced by oxygen stress, was upregulated. L-carnitine protects alveolar epithelial cells from apoptosis (Conlon et al., 2016) and also has a protective effect against lung damage (Yano et al., 2008; Tousson et al., 2014; Kaya et al., 2015; Conlon et al., 2016), and is reduced during emphysema progression. Psychosine and progesterone levels increased in the acute period, and may exacerbate the lung damage induced by influenza virus. Psychosine is a highly cytotoxic lipid, while progesterone is known to attenuate airway remodeling (Zhang et al., 2018). Shikimic acid, a precursor of oseltamivir phosphate and an inhibitor of the neuraminidase enzyme of influenza viruses (Martinez et al., 2015), decreased in the prodromal and acute phases. It is known to suppress the production of inflammatory cytokines by LPS (Rabelo et al., 2016), and can be used by bacteria for folate and aromatic amino acid biosynthesis. However, the relevance of low shikimic acid levels during influenza infection is still unknown. Carnosine treatment can decrease the mortality on account of H9N2 influenza virus infection, ameliorate pathological lesions in lungs (Xu et al., 2015), and accelerate repair in the injured lung (Perel’man et al., 1989). Increased carnosine levels in the recovery period of the infection alleviates the damage. Other differential metabolites which showed fold change >0.5 included cholesterol and D-Mannitol in the prodromal phase, and phosphocholine, hydroxyurea, phosphocreatine, hexylamine, thiamine, sebacic acid, DL-α-lipoic acid, FMN, acitretin, N-acetyl-L-histidine, dUDP, L-carnitine, L-threonine, and argininosuccinic acid in the acute period.

In conclusion, influenza virus infection induces a long-term alteration of LRT micro-environment, disrupts the LRT microbiome, alters the LRT metabolites and desensitizes the immune system of lung. The imbalance of homeostasis of LRT micro-environment may give a chance for the invasion of potential pathogens.

## Materials and Methods

### Animals and Samples Collection

Female C57BL/6 mice, aged 8 weeks, SPF, were purchased in one batch from Beijing Vital River Laboratory Animal Technology Co., Ltd. (Beijing, China). The mice were acclimatized for a week before the experiment under 12-h light/dark cycle, and fed *ad libitum*. Following deep anesthetization with 5% chloral hydrate (w/v, 400 mg/kg), the mice were inoculated with 50 μl saline/influenza (6.2 × 10^8^ PFU per mouse) virus via the intranasal route. The body weight was measured daily between 8 and 10 AM, and all activities were recorded. BALF and lung tissues were collected as described previously (Ferreira et al., 2017). Mice were euthanized by a lethal dose of 5% chloral hydrate (w/v, 600 mg/kg). The tracheas were cannulated with polyethylene tubing (24 G3/4). The lungs were then lavaged by instillation of 1 ml saline three times with total volume of 3 ml. The BALF was centrifuged at 1,500 rpm for 10 mins at 4°C, supernatant and pellets were separated and frozen at −80°C. The supernatant was used for metabolomic analysis, and the pellets were used for microbiome analysis. All animal experiments were approved by the Institutional Animal Ethics Committee of Shantou University Medical College, and performed under the guidelines of Administration of Affairs Concerning Experimental Animals.

### Extraction of Microbiota Genomic DNA

Lower respiratory tract samples (pellets of BALF) were collected on 1, 3, 7, 14, and 28 dpi. There were 5/6 mice in each group. Every sample was analyzed independently. Total genomic DNA was extracted using the CTAB/SDS method, and quantified by electrophoresing through 1% agarose gels. The DNA samples were diluted to 1 ng/μl using sterile water, and the 16S rRNA genes were amplified using barcoded primers (Phusion^®^ High-Fidelity PCR Master Mix, NEB, United States) specific for the V4 region (515F-806R) (Caporaso et al., 2012) and the Phusion^®^ High-Fidelity PCR Master Mix (NEB, United States).

### Microbiota Library Preparation and Sequencing

PCR products were run on 2% agarose gels, and the bands between 400 and 450 bp were sliced and purified with QIAquick Gel Extraction Kit (Qiagen, Germany). Sequencing libraries were generated using TruSeq^®^ DNA PCR-Free Sample Preparation Kit (Illumina, United States) according to the manufacturer’s instructions and index codes were added. The library quality was assessed on the Qubit^®^ 2.0 Fluorometer (Thermo Scientific, United States) and Agilent Bioanalyzer 2100 system, and sequenced on an Illumina HiSeq 2500 platform. Paired-end reads 250 bp in length were generated that were merged using FLASH (Magoc and Salzberg, 2011), and filtered by QIIME (v 1.7.0) (Caporaso et al., 2010; Bokulich et al., 2013). To obtain the effective tags, the raw tags were compared with the Gold database to detect and remove the chimera with the UCHIME algorithm (Edgar et al., 2011; Haas et al., 2011). The sequences were analyzed with the Uparse software (Uparse v7.0.1001) (Edgar, 2013), and those with ≥97% similarity were assigned to the same OTUs. Representative sequence for each OTU was screened for species annotation, and the taxonomic information was annotated based on the GreenGene database with RDP classifier (v 2.2). Phylogenetic relationship was analyzed with the MUSCLE software (v 3.8.31). The abundance of the OTUs were normalized using a standard sequence number corresponding to the sample with the fewest sequences.

### Sequence Analyses

The normalized data was used for alpha diversity and beta diversity analyses. Alpha diversity was calculated based on Observed-species, Chao1, Shannon, Simpson, ACE and Good-coverage indices with QIIME (Version 1.7.0), and displayed with R software (Version 2.15.3). Beta diversity on weighted uniFrac was calculated by QIIME software (Version 1.7.0). Cluster analysis was preceded by principal component analysis (PCA) to reduce the dimension of the original variables using the FactoMineR package and ggplot2 package in R software (Version 2.15.3). PCoA was then performed to obtain the principal coordinates and visualize them from complex, multidimensional data. A distance matrix of inter-sample weighted uniFrac was transformed to a new set of orthogonal axes, in which the maximum variation factor was demonstrated by the first principal coordinate, and the second maximum by the second principal coordinate etc. PCoA analysis was displayed by WGCNA package, stat packages and ggplot2 package in R software (Version 2.15.3). UPGMA hierarchical clustering was performed to interpret the distance matrix using average linkage using the QIIME software (Version 1.7.0).

### Transcriptome Sequencing

RNA was extracted from lung tissue collected at 3, 7, and 28 dpi (*n* = 3 per group) using Trizol (Invitrogen) as per the manufacturer’s instructions. The concentration of RNA was measured using Qubit^®^ RNA Assay Kit in Qubit^®^ 2.0 Fluorometer (Life Technologies, Camarillo, CA, United States), and its integrity was assessed using the RNA Nano 6000 Assay Kit of the Bioanalyzer 2100 system (Agilent Technologies, Santa Clara, CA, United States). Sequencing libraries were generated using NEBNext^®^ Ultra^TM^ RNA Library Prep Kit for Illumina^®^ (NEB, United States) according to the manufacturer’s instructions, and sample-specific index codes were added. Briefly, the mRNAs were purified from total RNA using poly-T oligo-attached magnetic beads, and fragmented using divalent cations at high temperature in the NEBNext First Strand Synthesis Reaction Buffer (5X). First strand cDNA was synthesized using random hexamer primers and M-MuLV Reverse Transcriptase (RNase H^–^), and the second strand using DNA polymerase I and RNase H. The remaining overhangs were converted into blunt ends using exonuclease/polymerase, and after adenylation of the 3′ ends, ligated to NEBNext Adaptors with hairpin loop structure. To enrich for 150–200 bp long cDNA sequences, the library fragments were purified with AMPure XP system (Beckman Coulter, Beverly, MA, United States). The size-selected, adaptor-ligated cDNA was incubated with 3 μl uracil-specific extension reagent (USER) enzyme (NEB, United States) at 37°C for 15 min, followed by 5 min at 95°C. PCR was performed with Phusion High-Fidelity DNA polymerase, Universal PCR primers and Index (X) Primer. The PCR products were purified using the AMPure XP system, and library quality was assessed on the Agilent Bioanalyzer 2100 system. The index-coded samples were clustered on a cBot Cluster Generation System using TruSeq PE Cluster Kit v3-cBot-HS (Illumina) according to the manufacturer’s instructions. The library preparations were then sequenced on an Illumina HiSeq platform and 125 bp/150 bp paired-end reads were generated.

Reference genome and gene model annotation files were downloaded from the web, and an index of the reference genome was built using Bowtie (v2.2.3). The paired-end clean reads were aligned to the reference genome using TopHat (v2.0.12), which can generate a database of splice junctions based on the gene model annotation file and provide a better mapping result compared to other non-splice mapping tools. HTSeq (v0.6.1) was used to count the reads numbers mapped to each gene, and the expected number of fragments per kilobase of transcript sequence per million base pairs sequenced or FPKM of each gene was calculated based on its length and mapped reads count (Trapnell et al., 2010).

### Differential Expression Analysis

For each sequenced library, the read counts were first adjusted by edgeR program package through one scaling normalized factor. The DEGs between two groups (three biological replicates per group) were identified using the DESeq R package (1.18.0) which uses a model based on the negative binomial distribution. The resulting *P*-values were adjusted using the Benjamini and Hochberg’s approach for controlling the false discovery rate (FDR), and the genes with an adjusted *P*-value <0.05 were considered DEGs.

The Gene Ontology (GO) enrichment analysis of DEGs was implemented using the GOseq R package, wherein the gene length bias was corrected. GO terms with corrected *P* value less than 0.05 were considered significantly enriched. Finally, the KOBAS software was used to test for statistical enrichment of the DEGs in the different KEGG pathways^1^.

### Metabolomics Analysis Based on LC/MS

Thirty-six samples were subjected to metabolomics analysis based on the LC/MS method. Briefly, BALF was collected from the inoculated mice at 3, 7, and 28 dpi (*n* = 6 per group), and centrifuged at 12,000 rpm for 30 min at 4°C. The supernatants were collected, and 100 μl of each sample was mixed with 300 μl methanol and 10 μl DL-o-Chlorophenylalanine. After centrifuging at 12,000 rpm for 15 min at 4°C, 200 μl of each supernatant was used for LC/MS.

LC was performed on the ACQUITYTM UPLC-QTOF system and Waters ACQUITY UPLC HSS T3 (2.1 mm × 100 mm × 1.8 um) column preheated at 40°C. The gradient conditions for metabolite elution are shown in Supplementary Table S2. The mobile phase was composed of water with 0.1% formic acid as solvent A, and acetonitrile with 0.1% formic acid as solvent B for positive ion mode (ESI+) and negative ion mode (ESI−).

MS parameters were as follows: capillary voltage −1.4 kV in ESI+ and 1.3 kV in ESI−, the flow rate of cone gas and desolvation gas −50–600 L/h, ion source temperature −120°C, desolvation temperature −350°C, ion energy −1 V, collision energy −10–40 V, and inter scan time −0.02 s. Masses ranging from 50 to 1500 ion mass (m/z) were acquired. The data, including retention time (RT), mass-to-charge ratio (MZ), observations (samples) and peak intensity, were extracted and preprocessed with XCMS in R software, and then normalized and edited into two-dimensional data matrix using Excel 2010 software. A total of 1379 and 1202 features were, respectively, collected at the ESI+ and ESI− ion modes. Multivariate analysis was performed using the SIMCA-P software (v 13.0, Umetrics AB, Umea, Sweden).

### Statistical Analysis

Body weight loss was compared using the unpaired *t* test in Graph Pad Prism (v7.0). Shannon indices were analyzed with Wilcox rank sum test, weighted uniFrac distances with non-parametric ANOSIM test, and correlation between samples with Pearson correlation test using R software.

## Data Availability Statement

The raw data supporting the conclusions of this manuscript will be made available by the authors, without undue reservation, to any qualified researcher.

## Ethics Statement

The animal study was reviewed and approved by Institutional Animal Ethics Committee of Shantou University Medical College.

## Author Contributions

LG performed the experiments, analyzed the results, and wrote the manuscript. HD and YZ recorded the bodyweight and collected the tissue samples. ZR, SY, YG, JD, XC, and KL performed the supporting experiments. GW and RL conceived and supervised the study and wrote the manuscript. All authors contributed to manuscript revision, read and approved the submitted version.

## Conflict of Interest

The authors declare that the research was conducted in the absence of any commercial or financial relationships that could be construed as a potential conflict of interest.

## Footnotes

1 http://www.genome.jp/kegg/
