# Supplementary material for: Dynamic Changes in the Microbiome and Mucosal Immune Microenvironment of the Lower Respiratory Tract by Influenza Virus Infection
Source: Front Microbiol. 2019 Nov 1;10:2491. doi: 10.3389/fmicb.2019.02491 (PMC6838016; doi:10.3389/fmicb.2019.02491)
Supplement: Supplementary file 1 [file Data_Sheet_1.PDF]

Figure S1. Rarefaction curves for gene number in samples (n=54). The curve is near smooth indicating reliable sequencing data (**C**, the group treated with saline; **F**, the group treated with influenza virus; the number followed **C**/**F** represented the day post inoculation).

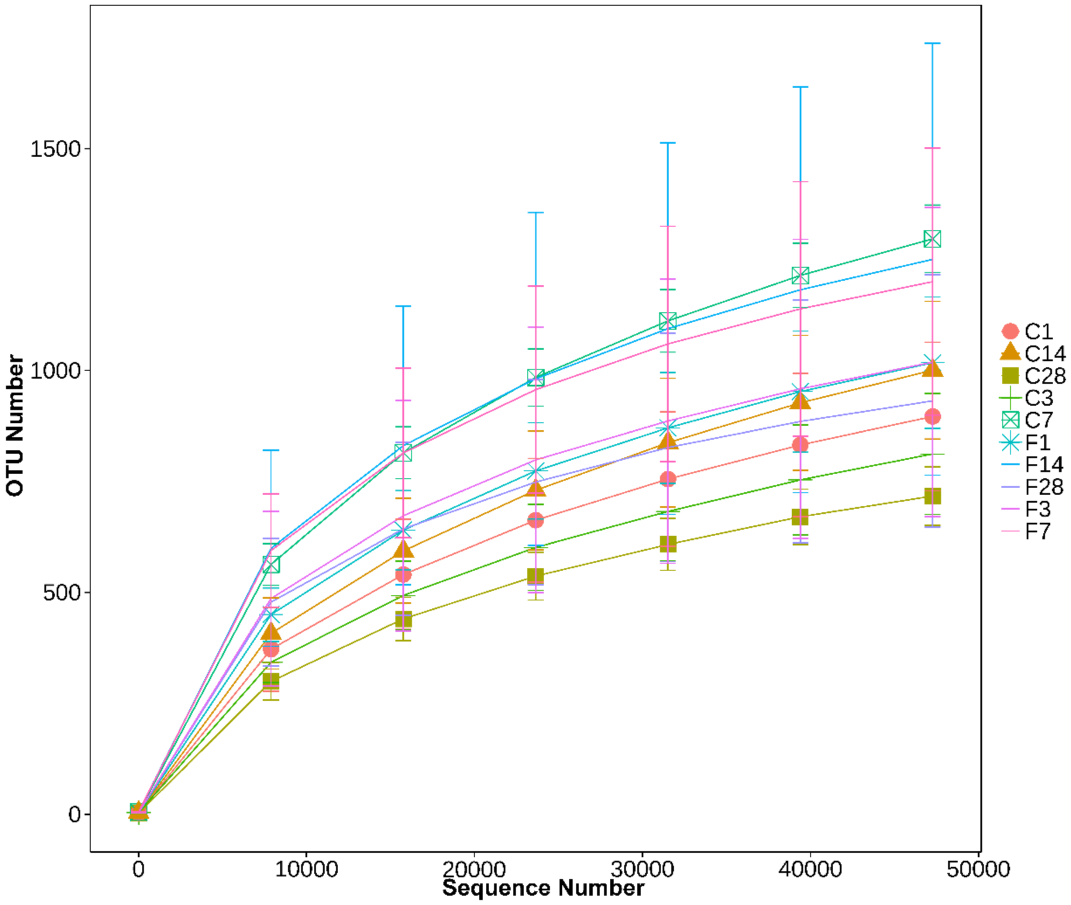

Figure S2. Principal Co-ordinates Analysis (PCoA) plot based on weighted unifrac distance (**C**, the group treated with saline; **F**, the group treated with influenza virus; the number followed **C**/**F** represented the day post inoculation)

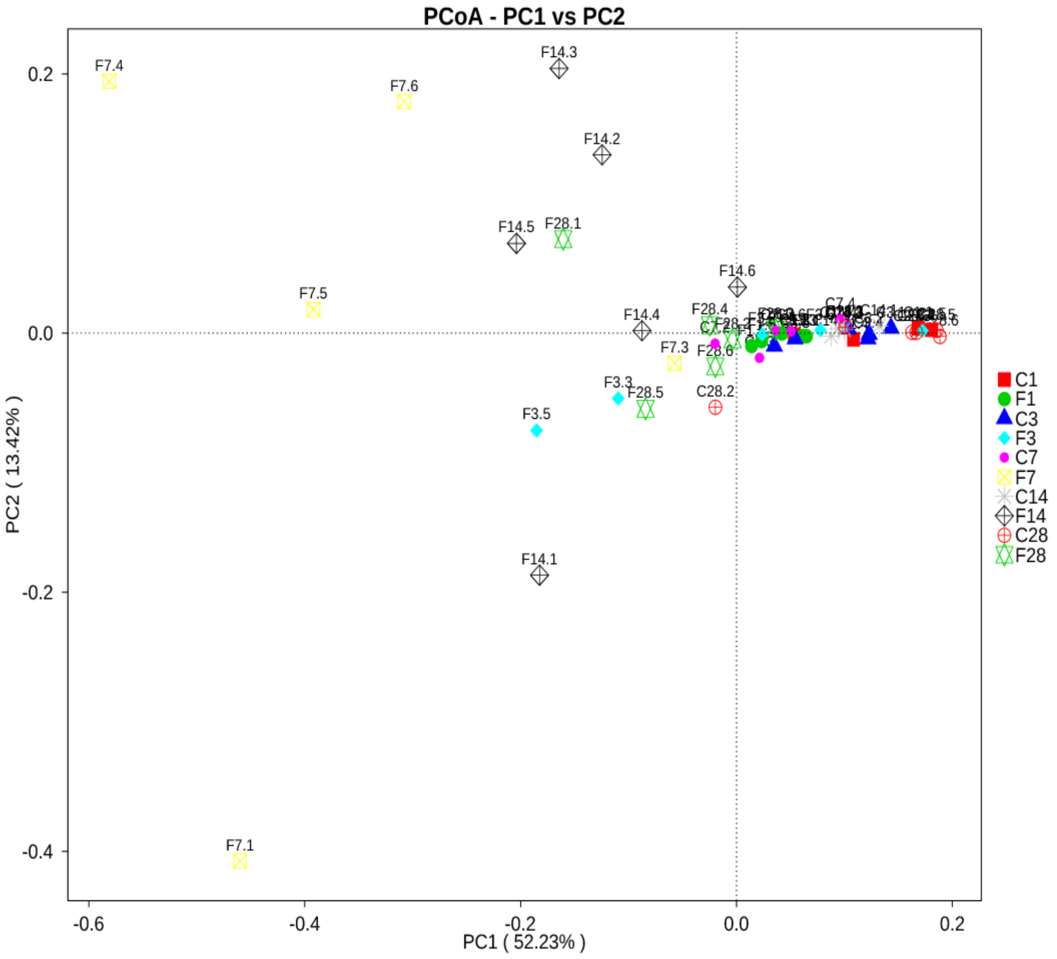

Figure S3. Heat map of Pearson correlation between samples. R^2 is the square of Pearson correlation coefficient (**C**, the group treated with saline; **F**, the group treated with influenza virus; the number followed **C**/**F** represented the day post inoculation; 3 replications per group).

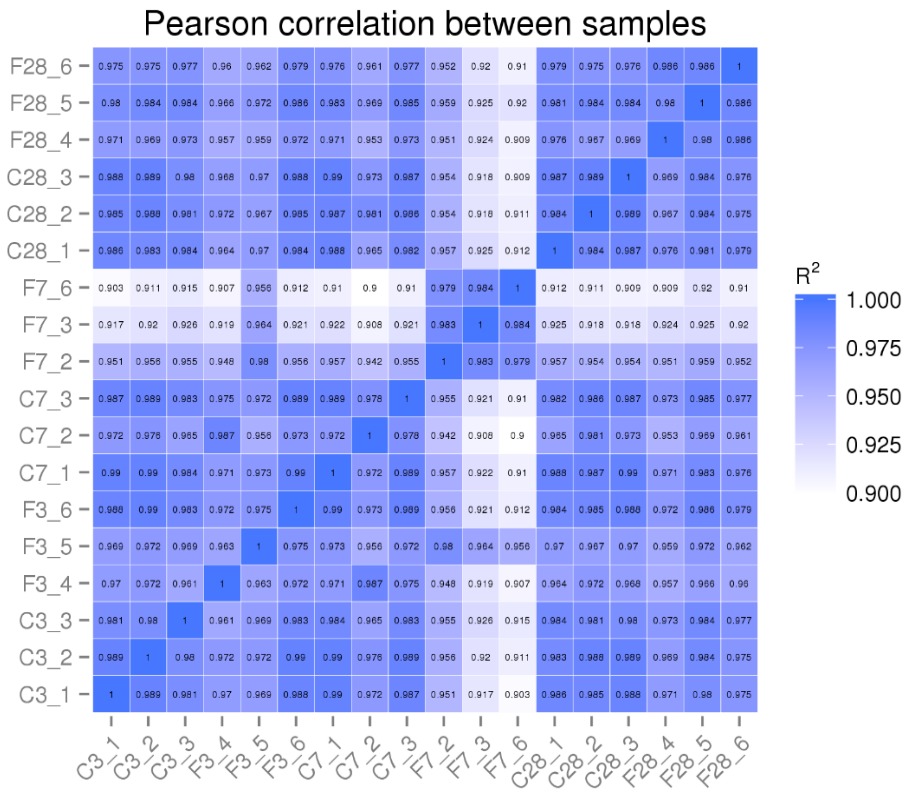

Figure S4. Correlation between the differential metabolites (left) and genomic function of microbiota (down) in the prodromal period (3 dpi), **(a)** positive model; **(b)** negative model.

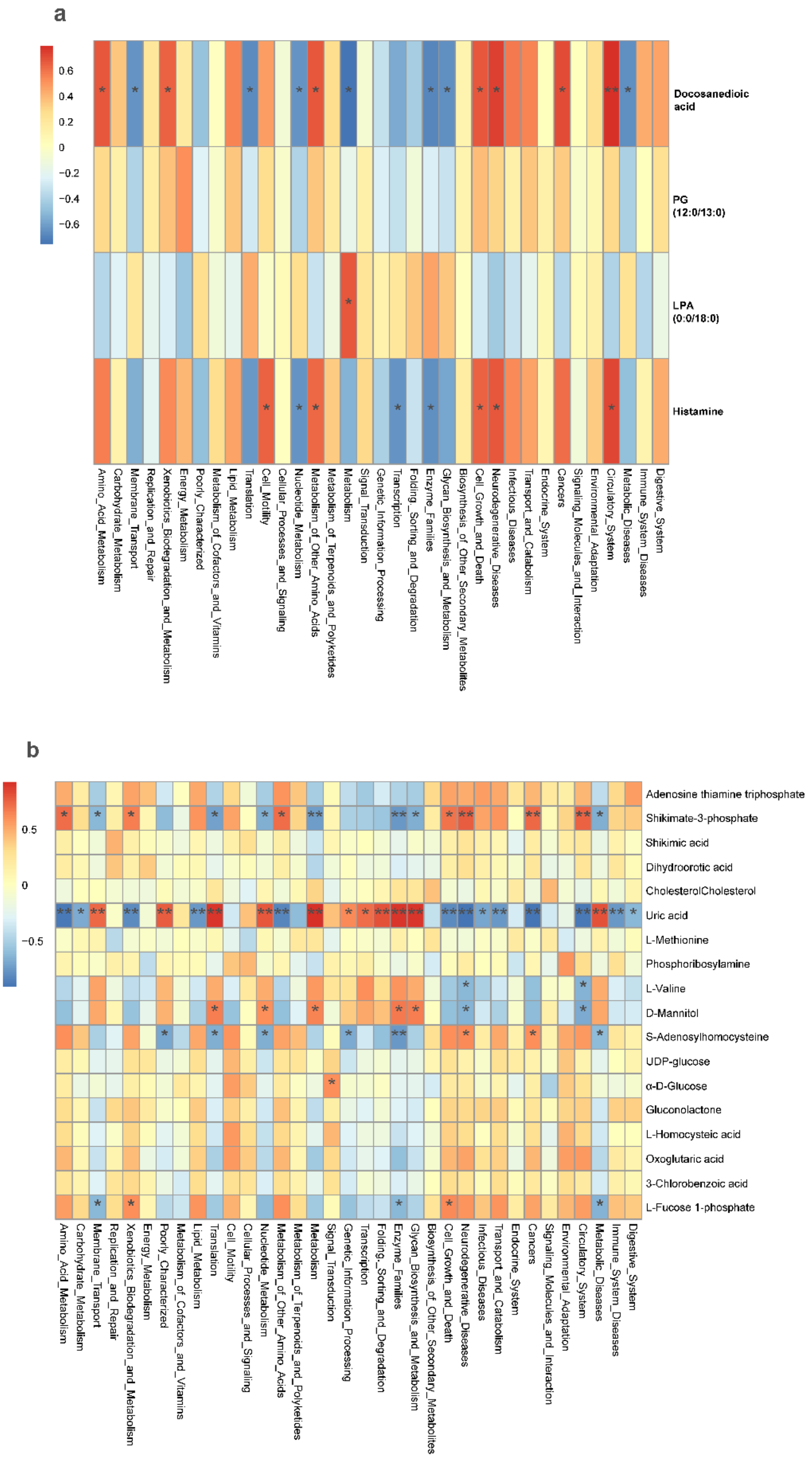

Figure S5. The correlation between the differential metabolites and genomic function of microbiota in the acute period (7 dpi)

a

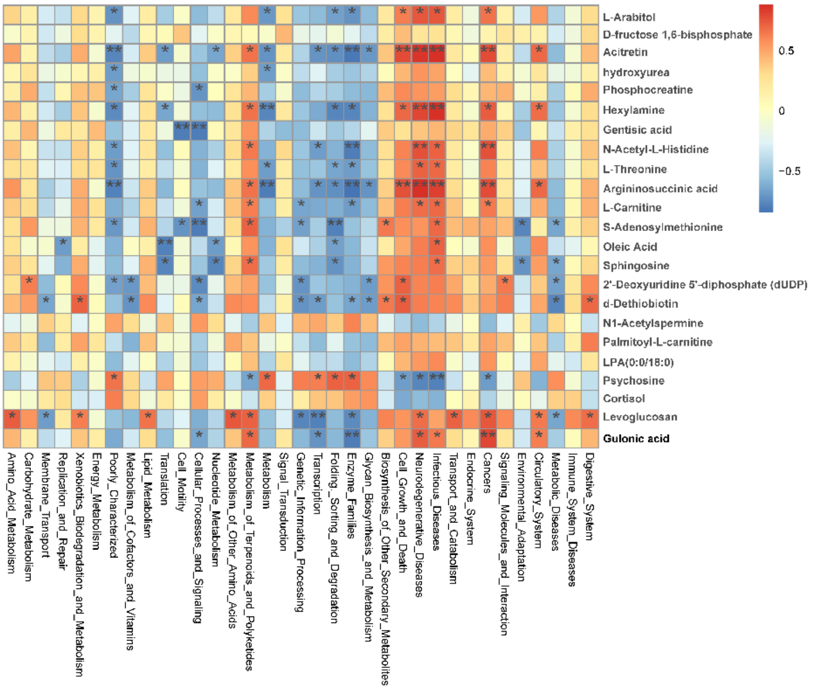

b

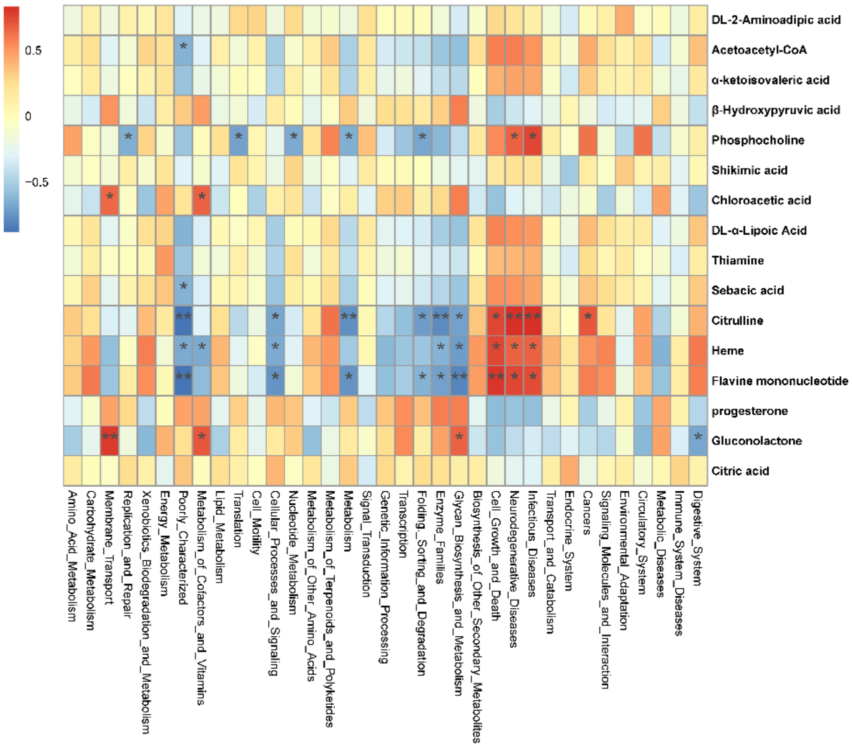

Figure S6. The correlation between the differential metabolites (left) and genomic function of microbiota (down) in the recovery period (28 dpi)

a

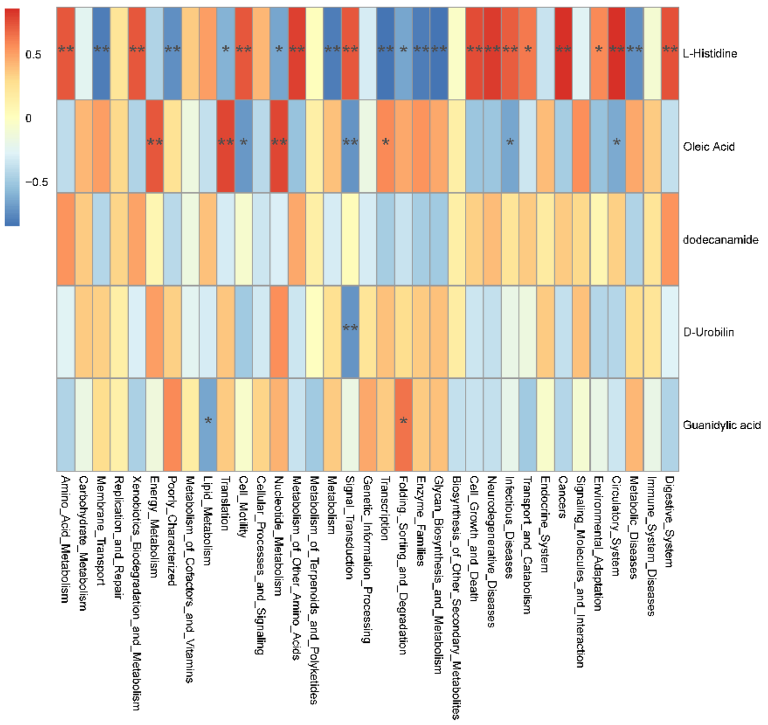

b

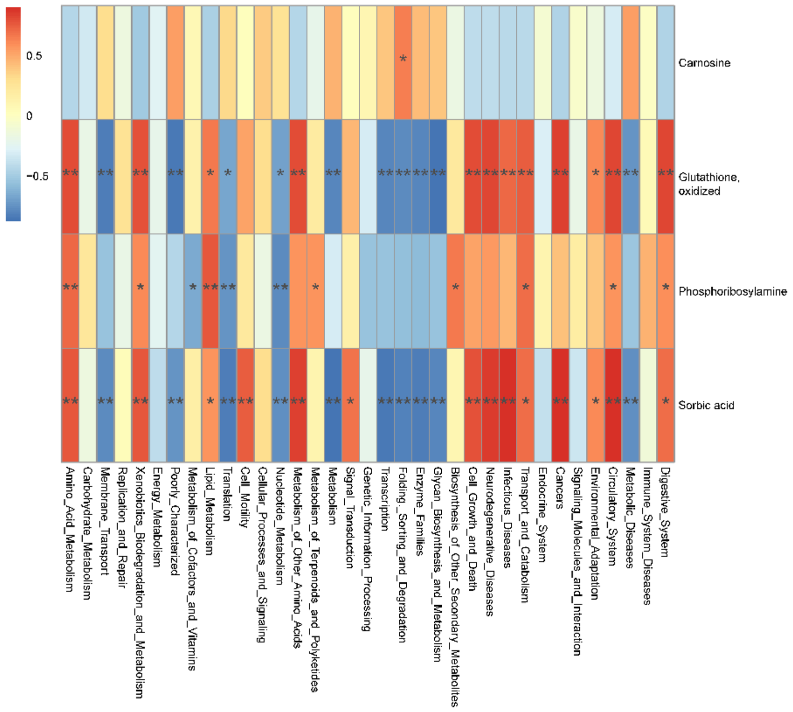

Table S1. Wilcox rank sum test for inter-group differences in Shannon index

Groups	Difference	LCL	UCL	*p* value	sig.
C1-F1	-6.8	-17.9957	4.395725	0.2274	NS
C3-F3	-16.3667	-27.0858	-5.64758	0.0036	**
C7-F7	-12.4	-23.5957	-1.20427	0.0307	*
C14-F14	-24.1333	-34.8524	-13.4142	p<0.001	***
C28-F28	-28.8333	-39.0536	-18.6131	p<0.001	***
F1-F3	-11	-22.1957	0.195725	0.054	NS
F1-F7	-28.6	-39.7957	-17.4043	p<0.001	***
F1-F14	-22.1333	-32.8524	-11.4142	0.0001	***
F1-F28	-14.8	-25.5191	-4.08091	0.0079	**
F3-F7	-17.6	-28.7957	-6.40427	0.0028	**
F3-F14	11.13333	0.414243	21.85242	0.0421	*
F3-F28	3.8	-6.91909	14.51909	0.4787	NS
F7-F14	-6.46667	-17.1858	4.252424	0.2305	NS
F7-F28	-13.8	-24.5191	-3.08091	0.0128	*
F14-F28	7.333333	-2.88692	17.55359	0.1552	NS
C1-C3	-1.43333	-12.1524	9.285757	0.7888	NS
C1-C7	-23	-34.1957	-11.8043	0.0002	***
C1-C14	-4.8	-15.9957	6.395725	0.3922	NS
C1-C28	7.233333	-3.48576	17.95242	0.1808	NS

Table S2. The gradient of mobile phase

Time（min）	Flow（ml/min）	Pressure Limit（bar）	Solv Ratio B（%）
0	0.35	800	5
1	0.35	800	5
6	0.35	800	20
9	0.35	800	50
13	0.35	800	95
15	0.35	800	95
